# Interaction of chemotherapy and radiotherapy in altering the shape of subcortical structures in patients with nasopharyngeal carcinoma

**DOI:** 10.3389/fonc.2022.952983

**Published:** 2022-09-12

**Authors:** Feibiao Nan, Jian-ming Gao, Li Li, You-ming Zhang, Yuanchao Zhang

**Affiliations:** ^1^ Yangtze Delta Region Institute (Huzhou), University of Electronic Science and Technology of China, Huzhou, China; ^2^ Key Laboratory for NeuroInformation of Ministry of Education, School of Life Science and Technology, University of Electronic Science and Technology of China, Chengdu, China; ^3^ Department of Radiation Oncology, Sun Yat-sen University Cancer Center, State Key Laboratory of Oncology in South China, Collaborative Innovation Center for Cancer Medicine, Guangzhou, China; ^4^ Sun Yat-sen University Cancer Center, State Key Laboratory of Oncology in South China, Collaborative Innovation Center for Cancer Medicine, Guangzhou, China; ^5^ Department of Radiology, Xiangya Hospital, Central South University, Changsha, China; ^6^ National Clinical Research Center for Geriatric Diseases, Xiangya Hospital, Central South University, Changsha, China

**Keywords:** Nasopharyngeal carcinoma, subcortical structures, radiotherapy, chemoradiotherapy, shape analysis, thalamus

## Abstract

Neuroimaging studies have found significant structural alterations of the cerebral cortex in patients with nasopharyngeal carcinoma (NPC) following radiotherapy (RT) or concomitant chemoradiotherapy (CCRT), while their effects on the shape of subcortical structures remain largely unknown. In this study, we investigated the subcortical shape alterations between three groups: 56 untreated NPC patients (pre-RT group), 37 RT-treated NPC patients (post-RT group), and 108 CCRT-treated NPC patients (post-CCRT group). Using FSL-FIRST, we found that, compared with the pre-RT group, the post-CCRT group exhibited significant inward atrophy in the bilateral thalamus, bilateral putamen, left pallidum, and left caudate and outward inflation in the left caudate, while the post-RT group only exhibited inward atrophy in the bilateral thalamus. In addition, greater maximum dosage of RT for temporal lobes was associated with more severe inward atrophy of the bilateral thalamus in treated NPC patients. These results indicated that there may be an interaction between RT and CT that can cause subcortical damage.

## Introduction

Nasopharyngeal carcinoma (NPC) is a malignancy arising from the nasopharynx epithelium. It is an endemic disease with a high incidence rate in east and southeast Asia (especially in southern China) (1). To date, radiotherapy (RT) has been considered the primary treatment modality for NPC (2). For patients with locally advanced NPC, chemotherapy (CT) can be applied alongside with RT. Despite the effectiveness of RT and CT in disease control (3,4), they are often associated with serious adverse effect on the central nervous system, leading to visual and auditory impairment, progressive memory loss, and increased negative emotions (depression, anxiety) (4–6). Moreover, it has been shown that concomitant chemo-radiotherapy (CCRT) is associated with more severe side effects on the brain than RT alone (7) or CT alone (8).

In recent years, advances in neuroimaging techniques have provided new and exciting avenues for us to noninvasively characterize the macroscopic changes of brain parenchyma *in vivo*. In NPC, most of previous neuroimaging studies were conducted attempting to examine the effects of RT on brain structure and function. For example, studies using structural MRI data have revealed that compared with patients before RT, patients with NPC have significantly decreased gray matter after RT, mainly involving the temporal lobe, hippocampus and the cerebellum, and that the gray matter decreases were dose- and time-dependent (3, 9–13). Using diffusion tensor imaging (DTI), studies have found that RT could decrease fractional anisotropy and increase mean diffusivity of the white matter in cerebellum, temporal lobe, frontal lobe and parietal lobe in patients with NPC (10, 14, 15). A DTI-based network study found that brain global network properties (e.g. clustering coefficient, normalized characteristic path length, normalized clustering coefficient) decreased in patients with NPC within 6 months after RT, compared with those without RT (16). Using resting-state fMRI, some studies have found that the cerebellum and some regions of default mode network, including the hippocampus, temporal lobe, and posterior cingulate cortex (PCC), were associated with altered functional connectivity to other regions in (17–19). Compared with patients with NPC who did not receive RT, RT-treated NPC patients had aberrant regional homogeneity (ReHo) values in cerebellum, temporal lobe and insula and aberrant ReHo values had different dynamic changes patterns over time (20). In contrast, less attention has been paid to the effect of CT on the brain. Using multimodal MRI data, our recent study investigated the effects of RT and CT on the cortical morphology and functional connectivity in patients with NPC, and found that chemotherapy potentially facilitated the occurrence of radiation encephalopathy in treated NPC patients (21). One question remains, however, as to whether RT and CT could affect the morphology of subcortical structures in patients with NPC.

In the present study, we aimed to investigate the effects of CT and RT on the shape of subcortical structures in patients with NPC. Specifically, we used structural MRI data to characterize the shape abnormalities of subcortical nuclei in a cohort of patients with NPC who had undergone RT (Post-RT) or CCRT (Post-CCRT), as compared to untreated NPC patients (Pre-RT). We hypothesized that, compared with Pre-RT group, Post-RT group would show significant abnormalities in the shape of the subcortical nuclei including the thalamus, while Post-CCRT group would show more extensive changes in the shape of the subcortical nuclei including the thalamus.

## Materials and methods

### Subjects

This retrospective cross-sectional study included 201 patients with pathologically diagnosed NPC from March 2011 to July 2015. They were divided into three groups according to the difference in treatment methods: the Pre-RT group (56 NPC patients who had not received treatment), the Post-RT group (37 NPC patients who had received RT) and the Post-CCRT group (108 NPC patients who had received CCRT) (21). To measure the effect of RT or CCRT on the structure of subcortical nuclei, other possible confounding factors (such as age, sex, time intervals between RT and sMRI examinations, RT technology, and maximum RT dosage to the temporal lobes) had to be evenly distributed among the groups. The inclusion criteria for all patients in this study were as follows: (1) Pathologically confirmed NPC patients; (2) normal-appearing brain parenchyma on MRI; (3) right-handedness; (4) more than 6 years of education; and (5) an age range from 20 to 60 years. The exclusion criteria for all patients in this study were as follows: (1) brain parenchymal invasion, (2) brain tumor, (3) prior substantial head trauma or surgery, (4) neurological or psychiatric illness, (5) alcoholism or drug abuse, (6) any other major intracranial disease (22).

For each NPC patient, the following clinical data were available: the Karnofsky Performance Status (KPS) score, the main side of NPC, clinical stage, RT techniques, maximum dosage of RT to the temporal lobes, time intervals between RT and MRI examination, detailed information on the chemotherapy agents. All patients receiving RT were given Intensity-modulated radiation therapy (IMRT) or conventional two-dimensional radiotherapy (2D-CRT), as shown in **Table 1** [see our previous work (21)]. All patients who presented with stage II-IV NPC according to the 7th edition of the UICC/AJCC (2009) TNM classification (T = Tumor, N = Nodes, and M = Metastasis) (23) received CCRT, either with or without neoadjuvant/adjuvant chemotherapy. Specifically, the CCRT dosage regimen was cisplatin (100 mg/m^2^ intravenously on day 1). Additional adjuvant chemotherapy regimen was 3 cycles of cisplatin (80 mg/m^2^ intravenously on day 1) and fluorouracil (4 g/m^2^ in continuous intravenous infusion over 120 h). Additional neoadjuvant chemotherapy regimen was two cycles of cisplatin (80 mg/m^2^ intravenously on day 1) and fluorouracil (4 g/m^2^ in continuous intravenous infusion over 120 h) (22).

**Table 1:** Clinical data of the participants.

Items	pre-RT	post-RT	post-CCRT	p
Age (years),(mean ± SD)	47.0 ± 9.0	48.5 ± 10.1	45.5 ± 7.8	0.17
Sex				
male	43 (21.4)	29 (14.4)	74 (36.8)	0.59
female	13 (6.5)	8 (4.0)	19 (9.5)	
Main side of NPC				
Left, n	17 (8.4)	8 (4.0)	20 (10.0)	0.51
Right, n	22 (10.9)	18 (9.0)	50 (24.9)	
Bilateral, n	17 (8.4)	11 (5.5)	38 (18.9)	
Clinical staging				
I/II, n	14 (7.0)	29 (14.4)	23 (11.4)	<0.01*
III/IV, n	42 (20.9)	8 (4.0)	85 (42.3)	
Time intervals between RT and MRI examinations (month)	NA	20.9 ± 25.4	16.9 ± 23.8	0.39
RT technology				
IMRT, n	NA	28 (19.3)	86 (59.3)	0.61
2D-CRT, n	NA	9 (6.2)	22 (15.2)	
KPS score, (median ± IQR, range)	90 ± 0,	90 ± 0,	90 ± 0,	0.59
	80-90	80-90	80-90	
Maximum dosage of RT for temporal lobes (Gy)				
Left	NA	62.6 ± 7.8^a^	66.0 ± 10.3^b^	0.14
Right	NA	64.6 ± 7.0^a^	67.5 ± 8.1^b^	0.13

SD, standard deviation; UICC, International Union against Cancer; AJCC, American Joint Committee on Cancer; RT, radiation therapy; IMRT, intensity-modulated radiation therapy; 2D-CRT, conventional two-dimensional radiotherapy; KPS, Karnofsky Performance Status; IQR, interquartile range. (1) Data in parentheses are percentages; (2) The superscripts a and b denote data loss in 5 and 9 subjects respectively. *P<0.05.

### MRI acquisitions

All MRI data, including axial T1-weighted images, T2-weighted images, and T2-weighted fluid attenuated inversion recovery images for detecting any clinically silent lesions, were acquired on a Siemens Magnetom Tim Trio 3.0-T MR scanner with a 32-channel head coil. For each patient, high-resolution structural images were obtained using a T1-weighted 3D magnetization-prepared rapid acquisition gradient-echo sequence with 176 sagittal slices. The setting of scanning parameters in this study was as follows: repetition time (TR) = 2,300ms, echo time (TE) = 2.98ms, field of view (FOV) = 256 × 256mm, matrix size = 256 × 256, thickness/gap = 1.0/0mm, flip angle = 9°, and voxel size = 1.0 × 1.0 × 1.0mm (21, 22). During scanning, each subject was asked to lie motionlessly, close eyes and remain as equanimous as possible.

### Data processing and statistical analyses

All MRI data were analyzed with the software package FSL-FIRST (Analysis Group, FMRIB, Oxford, UK), a deformable-model-based active appearance model in a Bayesian framework (24). Our images were automatically segmented by applying the manually labelled information in the training images to the model training. The specific process of constructing the model is as follows. First, linear subcortical registration was applied in all images to achieve the correct alignment between the model and images. Second, while maintaining cross-subject vertex correspondence, a deformable 3D mesh was used to fit the training images to obtain manually labelled information, and then iteratively update its vertex position. Third, appearance was modeled with normalized intensity, which was to sample each training intensity image along the surface normal of the mesh vertex. Finally, the variation in the mesh fitting process for each image was modelled by vertex coordinates and intensity samples. Before the new image was fitted with the model, the model was registered into the native space using inverse transformation, and then the optimal fitting was obtained by maximizing the posterior probability. In this way, for each subject, we can obtain a surface mesh consisting of vertices and triangles. Because the subcortical surface meshes of all subjects were isomorphic, the comparisons between groups could be performed by examining group differences in the spatial location of each vertex. Although the vertices were in correspondence, the surface mesh was not in standard space, but in its native space. Before investigating group differences, the pose differences were removed by rigid alignment of the mean surface mesh in standard space, which meant that the sum-of-squares between a subject’s surface mesh and the mean surface mesh were minimized. After that, multivariate F-tests were performed for each vertex separately to detect between-group localized shape difference. Vertex-wise correlation analysis was also performed between the shape of subcortical nuclei and the maximum dosage of RT for temporal lobes in treated NPC patients. False discovery rate (FDR) theory was used to correct for multiple comparisons.

## Results

**Figure 1:**
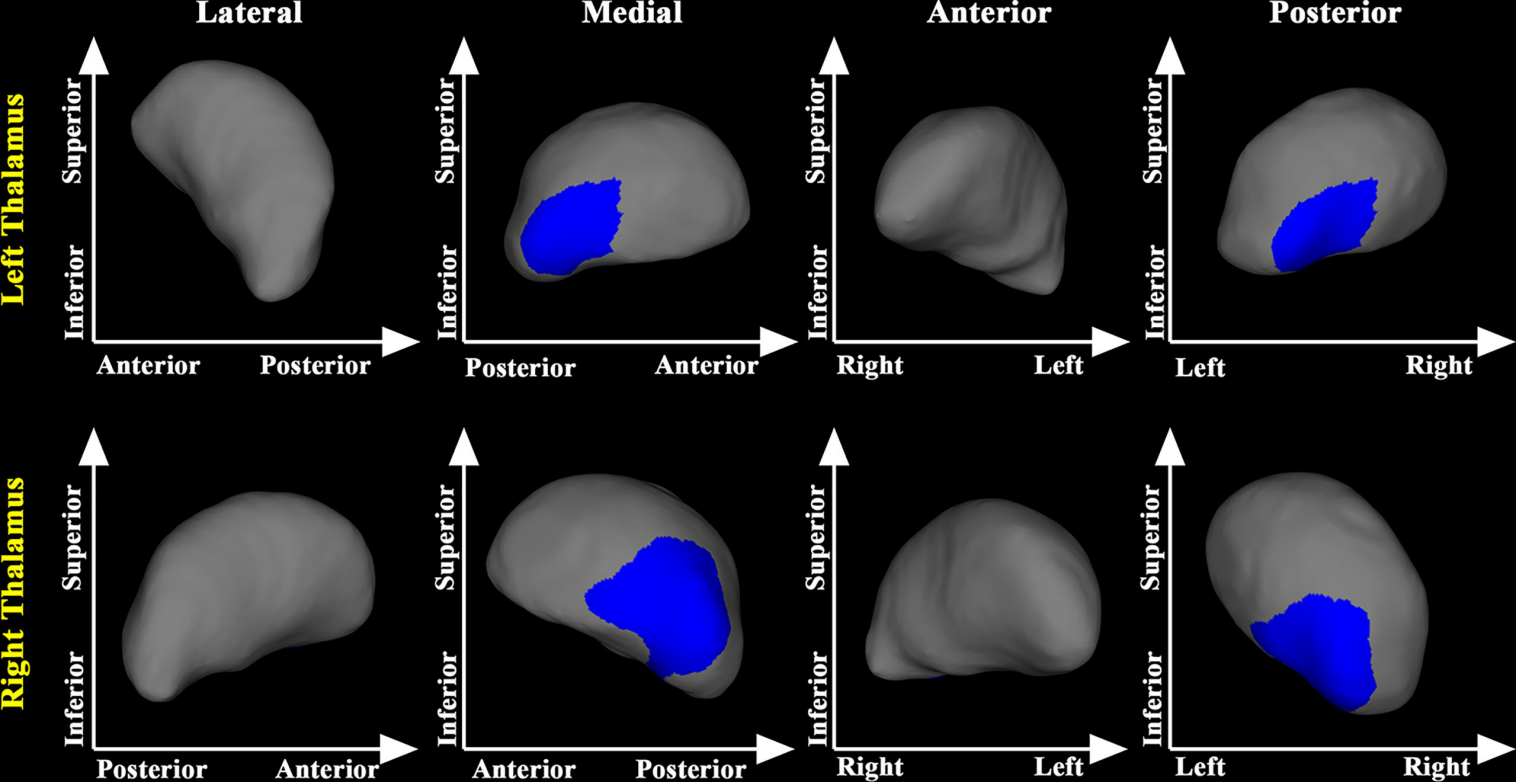
Thalamic shape differences between the post-RT and the pre-RT groups. The post-RT group showed significant thalamic atrophy compared with the pre-RT group. The blue color denotes regions with atrophy in the post-RT group compared with the pre-RT group.

Comparisons of the vertex locations between the Pre-RT group and the Post-RT group showed that Post-RT group exhibited significant regional atrophy in the medial posterior segment of the bilateral thalamus (**Figure 1**).

**Figure 2:**
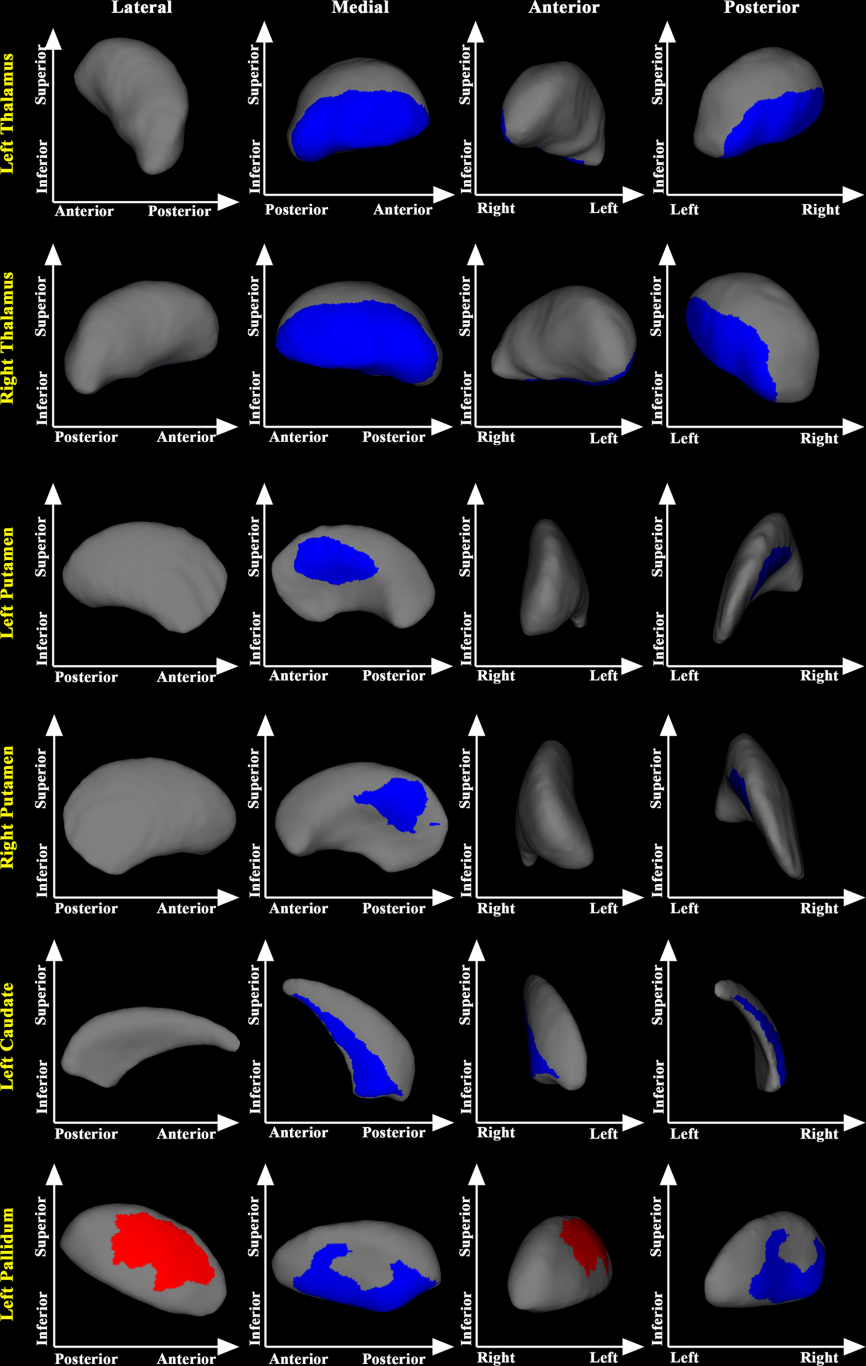
Subcortical shape differences between the post-CCRT and pre-RT groups. group compared with the pre-RT group. The post-CCRT group showed significant regional atrophy in bilateral thalamus, bilateral putamen, left caudate, and the left pallidum compared with the pre-RT group. The post-CCRT group also showed significant regional inflation in the left pallidum compared with the pre-RT group. The blue color denotes regions with atrophy in the post-CCRT group compared with the pre-RT group, while the red color denotes regions with inflation in the post-CCRT group compared with the pre-RT group.

Similarly, compared with the Pre-RT group, the medial posterior segment of the bilateral putamen, bilateral thalamus, left pallidum, and the left caudate were substantially atrophied in the Post-CCRT group. Further, the Post-CCRT group showed a significant regional inflation in the lateral anterior segment of the left pallidum (**Figure 2**).

**Figure 3:**
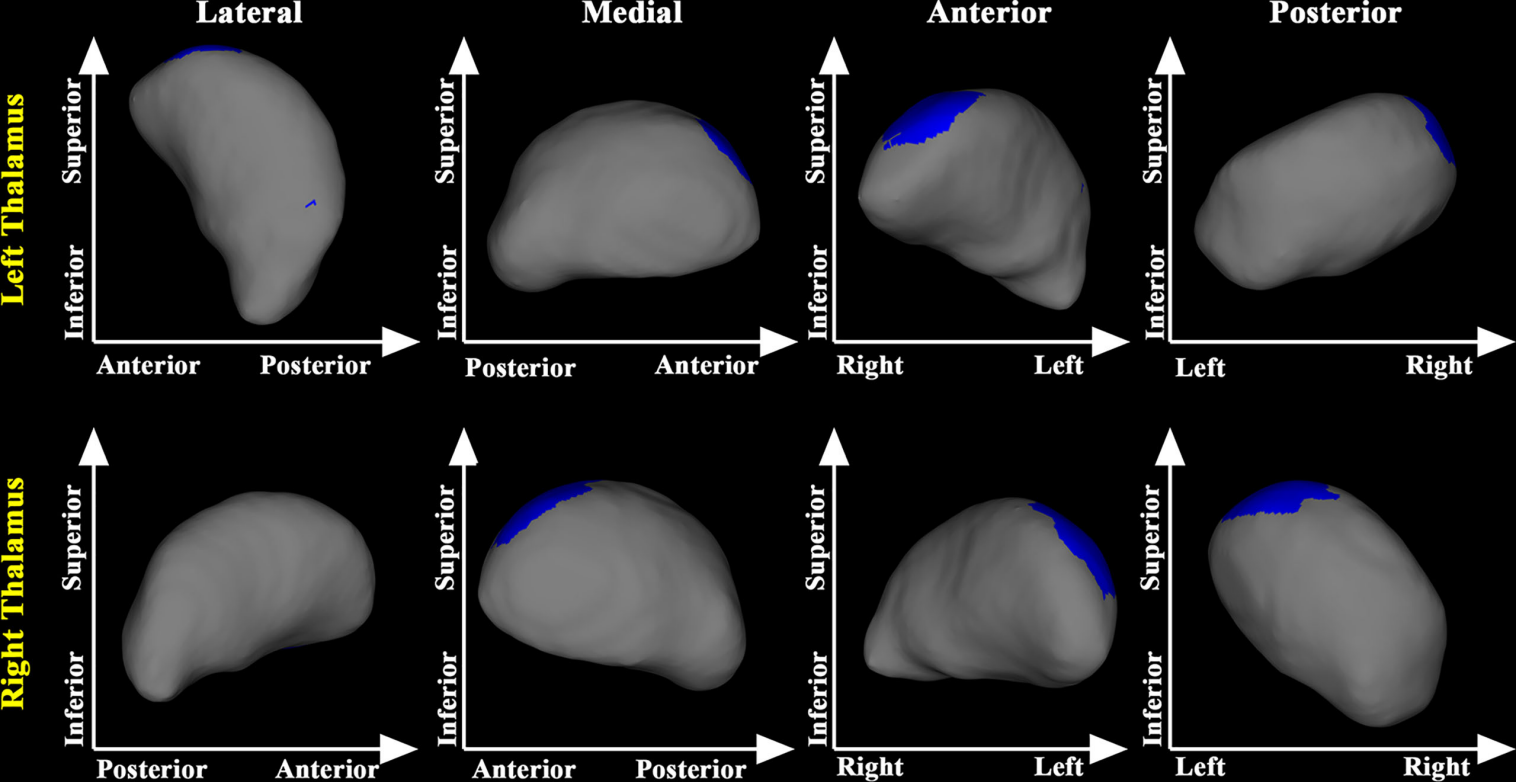
Relationship between maximum dosage of RT and morphology of the thalamus in treated NPC patients. Greater maximum dose of RT for temporal lobes was associated with more severe inward atrophy in the posterior part of the bilateral thalamus in treated NPC patients.

In the post-treatment patient groups (including post-RT and post-CCRT groups), greater maximum dosage of RT for temporal lobes was associated with more severe inward atrophy of the superior segment of the bilateral thalamus (**Figure 3**).

## Discussion

In this study, we examined the subcortical shape abnormalities in patients with NPC who underwent RT or CCRT, compared with untreated NPC patients. Our main findings can be summarized as follows. First, compared with the pre-RT group, patients in the post-RT group showed significant regional atrophy in the medial posterior segment of the bilateral thalamus. Second, compared with the pre-RT group, patients in the post-CCRT group showed significant regional atrophy in the medial posterior segment of the bilateral thalamus, bilateral putamen, left pallidum, and left caudate, and significant regional inflation in the lateral anterior segment of the left pallidum. Finally, in treated NPC patients, greater maximum dosage of RT for temporal lobes was associated with more severe inward atrophy of the superior segment of the bilateral thalamus. Taken together, these findings suggest that RT and CT may interact in altering the shape of subcortical structures in NPC.

Compared with the pre-RT group, patients in the post-RT group showed significant regional atrophy in the medial posterior segment of the bilateral thalamus. This finding is consistent with some previous fMRI studies, showing decreased fractional amplitude of low frequency fluctuation and ReHo values in the thalamus of NPC patients after RT (18, 25). The observed regional atrophy in the bilateral thalamus might be the anatomical substrates of the functional alterations in NPC patients treated with RT. Compared with the pre-RT group, patients in the post-CCRT group showed significant atrophy in the medial posterior segment of bilateral thalamus. Of note, the thalamic area identified with significant regional atrophy in the post-CCRT group was much larger than that identified in the post-RT group, suggesting possible interactions between RT and CT. In addition, using the pooled data of the post-RT and post-CCRT groups, we also found that greater maximum dosage of RT for temporal lobes was associated with more severe inward atrophy of the thalamus, suggesting that the thalamic abnormalities could arise from the direct effects of RT or from abnormal afferent inputs from RT-lesioned temporal lobe *via* the reciprocal thalamo-cortical connections (26).

Compared with the pre-RT group, patients in the post-CCRT group showed significant atrophy in the medial posterior segment of the bilateral putamen, left pallidum, and the left caudate. This finding is consistent with previous neuroimaging studies conducted to investigate the effect of CT on the brain structure. For example, Nelson et al. found higher mean diffusivity values in multiple subcortical nuclei (thalamus, putamen, globus pallidus, etc.) in long-term survivors of childhood brain tumor after CT (27). Similarly, two cross-sectional morphologic MRI studies showed significant reduction in the volume of multiple subcortical nuclei in long-term survivors of childhood acute lymphoblastic leukemia after CT (28, 29). Compared with the pre-RT group, the present study also found significant regional inflation in the lateral anterior segment of the left pallidum in the post-CCRT group. The finding of concurrent atrophy and inflation in the pallidum may indicate functional heterogeneity of different nuclei of the pallidum. However, the underlying mechanism of such alteration remains unclear and requires further investigation. Collectively, given that no significant shape abnormalities in putamen, pallidum and caudate were found in the post-RT group, we therefore speculate that the shape alterations of these subcortical structures may primarily occur as a result of CT.

The exact pathophysiological mechanism that leads to these significantly shape alterations of subcortical structures remains unclear. It is possible that multiple mechanisms, including DNA damage, apoptosis, inflammatory response and oxidative stress, vessel abnormity, and destruction of the blood-brain barrier (BBB), are involved in the development of the brain injury after RT or CT (30, 31). In fact, in the studies of the mechanism of RT or CT, the inflammatory response mediated by reactive microglia has always been of particular interest. More specifically, cellular debris from RT- or CT- damaged neuron could activate microglia, which in turn could release pro-inflammatory cytokines and other neurotoxic factors that damage neurons (32). Similarly, there is evidence that reactive astrocytes induced by reactive microglia are also neurotoxic (33). Besides, increased BBB permeability caused by CCRT also seems to be able to indirectly exacerbate existing levels of inflammatory response (34). However, we cannot exclude the involvement of other mechanisms in causing the subcortical shape alterations in treated NPC patients.

Functionally, the thalamus, as a hub, plays a particularly critical role in controlling the transmission and integration of information flow in the cortical and subcortical networks, which is the basis for its support of related cognitive functions (26, 35, 36). The thalamus and its connections to other brain regions (including the medial temporal lobe, cerebellum, prefrontal cortex, etc.) are instrumental in several neurocognitive domains, including memory, executive function, sleep and arousal, and information processing speed (26, 36–40), which are thought to be most affected by RT or CT (31, 41). Similarly, the putamen, the pallidum and the caudate have been involved in learning, language and reward mechanism, which are closely related to cognitive function (42–45). The shape alterations of these structures may be related to the neurocognitive changes after RT or CCRT for NPC patients. Alternatively, as the basal ganglia-thalamocortical circuits have been shown to be essential for motor control (46), alterations in these subcortical structures may also underlie the motor deficits in treated NPC patients, such as dysphagia (47).

## Limitation

There are some limitations for this study. First, this was a cross-sectional study. We cannot draw definitive conclusions about the causal mechanisms of subcortical shape abnormalities. Future longitudinal studies with a larger sample size are needed to address this problem. Second, due to the lack of NPC patients treated with CT alone, we cannot determine the exact role of CT in subcortical shape abnormalities. Third, the absence of detailed evaluations of psychological status, cognitive functions, and clinical signs and symptoms of patients, undermines the interpretation of our findings. Fourth, the specific effects of different chemotherapeutic agents on the shape of subcortical structures were not examined due to a lack of detailed dosage information.

## Conclusion

In this study, we investigated subcortical shape alterations in the post-RT and post-CCRT groups compared with the pre-RT group. We found that, compared with the pre-RT group, the post-CCRT group exhibited shape alterations in the bilateral thalamus, bilateral putamen, left pallidum, and left caudate while the post-RT group was only in the bilateral thalamus, suggesting that there may be an interaction between RT and CT for subcortical structural injury. Our findings provide new insights into the optimization of treatment options for NPC.

## Data availability statement

The raw data supporting the conclusions of this article will be made available by the authors, without undue reservation.

## Ethics statement

The studies involving human participants were reviewed and approved by Medical Research Ethics Committee of Xiangya Hospital, Central South University. The patients/participants provided their written informed consent to participate in this study.

## Author contributions

Y-mZ, LL and YZ conceived and designed the experiments. Y-mZ, LL, FN and YZ analyzed the data. YZ, FN, J-mG and YZ contributed materials and analysis tools. Y-mZ, FN, and YZ wrote the paper. All authors contributed to the article and approved the submitted version.

## Funding

This study was supported in part by the National Natural Science Foundation of China, Grant/Award Numbers: 82001784; The Natural Science Foundation (Youth Science Foundation Project) of Hunan Province, Grant/Award Numbers: 2021JJ41054; The Youth Science Foundation of Xiangya Hospital, Grant/Award Number: 2019Q16, and the Project of Science and Technology Department of Sichuan Province (grant number 2021YFS0240).

## Acknowledgments

The authors would like thank all the study participants for their efforts and enthusiasm for our clinical research. All authors reviewed the manuscript before submission.

## Conflict of interest

The authors declare that the research was conducted in the absence of any commercial or financial relationships that could be construed as a potential conflict of interest.

## Publisher’s note

All claims expressed in this article are solely those of the authors and do not necessarily represent those of their affiliated organizations, or those of the publisher, the editors and the reviewers. Any product that may be evaluated in this article, or claim that may be made by its manufacturer, is not guaranteed or endorsed by the publisher.
